# Dietary Salt Accelerates Orthodontic Tooth Movement by Increased Osteoclast Activity

**DOI:** 10.3390/ijms22020596

**Published:** 2021-01-09

**Authors:** Agnes Schröder, Joshua Gubernator, Alexandra Leikam, Ute Nazet, Fabian Cieplik, Jonathan Jantsch, Patrick Neubert, Jens Titze, Peter Proff, Christian Kirschneck

**Affiliations:** 1 Department of Orthodontics, University Hospital Regensburg, 93053 Regensburg, Germany; joshua.gubernator@stud.uni-regensburg.de (J.G.); alexandra.leikam@stud.uni-regensburg.de (A.L.); ute.nazet@ukr.de (U.N.); peter.proff@ukr.de (P.P.); christian.kirschneck@ukr.de (C.K.); 2 Department of Conservative Dentistry and Periodontology, University Hospital Regensburg, 93053 Regensburg, Germany; fabian.cieplik@ukr.de; 3 Institute of Clinical Microbiology and Hygiene, University Hospital Regensburg, 93053 Regensburg, Germany; jonathan.jantsch@ukr.de (J.J.); patrick.neubert@ukr.de (P.N.); 4 Cardiovascular and Metabolic Disease Programme, Duke-National University of Singapore, Singapore 169857, Singapore; jens.titze@duke-nus.edu.sg

**Keywords:** salt, orthodontic tooth movement, osteoclast activity

## Abstract

Dietary salt uptake and inflammation promote sodium accumulation in tissues, thereby modulating cells like macrophages and fibroblasts. Previous studies showed salt effects on periodontal ligament fibroblasts and on bone metabolism by expression of nuclear factor of activated T-cells-5 (NFAT-5). Here, we investigated the impact of salt and NFAT-5 on osteoclast activity and orthodontic tooth movement (OTM). After treatment of osteoclasts without (NS) or with additional salt (HS), we analyzed gene expression and the release of tartrate-resistant acid phosphatase and calcium phosphate resorption. We kept wild-type mice and mice lacking NFAT-5 in myeloid cells either on a low, normal or high salt diet and inserted an elastic band between the first and second molar to induce OTM. We analyzed the expression of genes involved in bone metabolism, periodontal bone loss, OTM and bone density. Osteoclast activity was increased upon HS treatment. HS promoted periodontal bone loss and OTM and was associated with reduced bone density. Deletion of NFAT-5 led to increased osteoclast activity with NS, whereas we detected impaired OTM in mice. Dietary salt uptake seems to accelerate OTM and induce periodontal bone loss due to reduced bone density, which may be attributed to enhanced osteoclast activity. NFAT-5 influences this reaction to HS, as we detected impaired OTM and osteoclast activity upon deletion.

## 1. Introduction

Orthodontic tooth movement (OTM) is based on multicellular processes and is characterized by remodeling of the periodontal ligament and alveolar bone [1] due to the activity of bone-resorbing osteoclasts and bone-forming osteoblasts [2]. In contrast to osteoblasts, which are derived from mesenchymal stem cells [3], osteoclasts evolve from hematopoietic stem cells [4]. Therefore two factors are critically involved: the macrophage-colony-simulating factor (M-CSF) and receptor activator of NF-kB ligand (RANKL), which promotes the differentiation of osteoclast precursor cells to bone-resorbing osteoclasts [5]. The binding of RANKL to the RANK receptor on precursor cells is strictly controlled by the decoy receptor osteoprotegerin (OPG) [6,7]. The periodontal ligament is a fibrous connective tissue, which anchors the teeth in the alveolar bone and contains fibroblasts [1], which are the main cell population, as well as immune cells such as macrophages and T cells [8,9]. Periodontal ligament fibroblasts play a major regulating role during orthodontic tooth movement, not only by secretion of inflammatory cytokines and chemokines, but also by secretion of RANKL and OPG [10,11,12].

Contrary to the guidelines of the World Health Organization (WHO), the dietary salt consumption of most people in Western countries is still around twice as high as recommended [13] and is associated with a wide range of diseases such as hypertension and osteopenia [14,15,16]. Sodium and chloride are the chemical components of salt. Sodium is the main cation within the extracellular fluid and is essential for the maintenance of normal cell function [17]. To date, there is evidence that local sodium content changes in various tissues in response to dietary salt consumption [16,18,19] and inflammatory processes [20], thereby influencing the activity of immune cells such as macrophages [16,20,21] and T-cells [22] and impairing osteoclastogenesis [19]. Therefore it can be assumed that sodium content in the periodontal ligament could influence the sterile pseudo-inflammatory reaction induced by orthodontic tooth movement in the periodontal ligament [10]. Most effects of salt are mediated by the transcription factor nuclear factor of activated T cells 5 (NFAT-5), which is responsible for the induction of transcription of osmoprotective genes [21,23,24].

Fibroblasts in the periodontal ligament increase the release of RANKL upon compressive force, thereby promoting OTM via increased osteoclastogenesis in pressure zones of the periodontal ligament [18,25]. Salt elevated this pressure-induced RANKL secretion by PDL fibroblasts in an in vitro study, indicating increased osteoclastogenesis [18]. Dietary salt treatment was shown to reduce bone density in the tibia, which also hints at an acceleration of OTM via increased osteoclastogenesis [19]. In this study we thus investigated the effects of salt on osteoclast activity in vitro and performed animal experiments to assess the role of dietary salt on the extent of OTM in vivo.

## 2. Results

### 2.1. Impact of Salt on Osteoclast Activity

**Figure 1:**
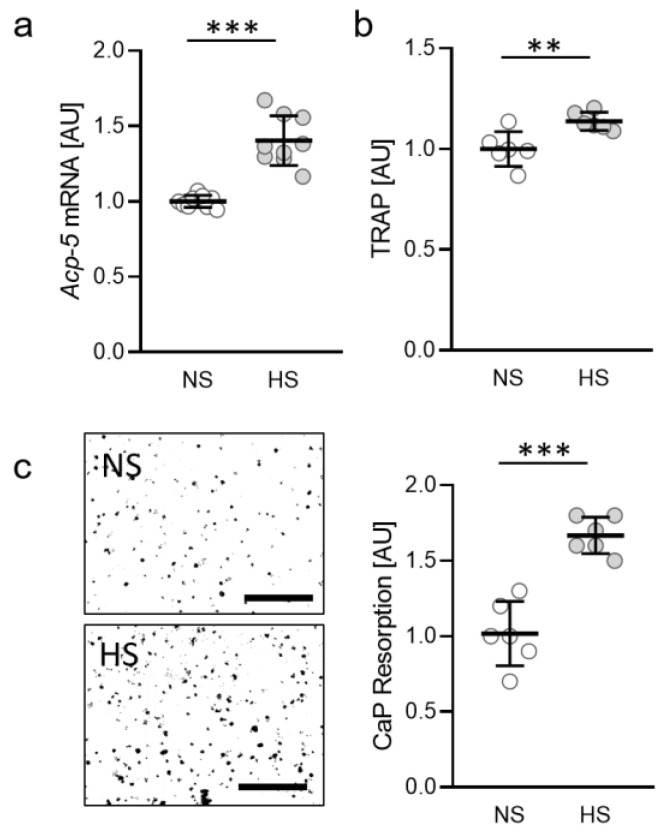
Gene expression of *Acp-5* (**a**), release of TRAP (**b**) and CaP resorption (**c**) with normal salt (NS) and high salt treatment (HS) in murine osteoclasts differentiated from RAW264.7 macrophages (n ≥ 6). Differentiation of osteoclasts was induced by treatment with M-CSF (30 ng/mL) and RANKL (50 ng/mL) for five days, followed by addition of 40 mM NaCl to the HS group. Symbols represent single data points, horizontal lines the arithmetic mean and vertical lines the standard error of the mean. AU: arbitrary units. Statistics: Two-tailed unpaired t-test. ** *p* < 0.01; *** *p* < 0.001.

First, we investigated the impact of salt (sodium chloride, NaCl) on gene expression of *tartrate-resistant alkaline phosphatase 5* (*Acp-5*) in osteoclasts differentiated from RAW264.7 macrophages. Under high salt conditions (HS; +40 mM NaCl) *Acp-5* gene expression was significantly elevated (*p* < 0.0001; Figure 1a). Accordingly, we measured increased amounts of tartrate-resistant alkaline phosphatase 5 (TRAP) in the cell culture supernatant (*p* = 0.0062; Figure 1b). To further investigate the activity of osteoclasts under HS conditions, we tested for calcium phosphate (CaP) resorption and detected enhanced resorption with HS (*p* < 0.0001; Figure 1c), indicating that HS conditions favor enhanced osteoclast activity.

### 2.2. Impact of NaCl-Containing Diets on Expression of Genes Involved in Bone Remodelling

**Figure 2:**
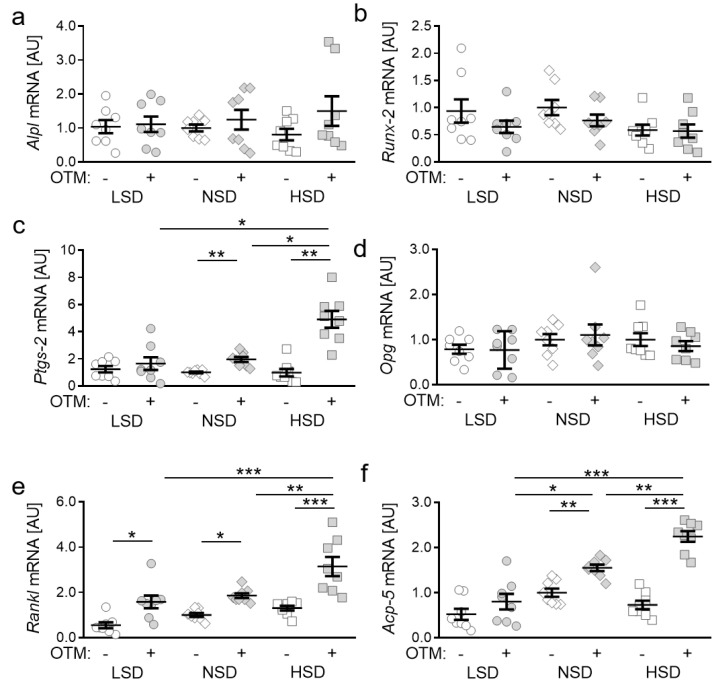
Gene expression of *Alpl* (**a**), *Runx-2* (**b**), *Ptgs-2* (**c**), *Opg* (**d**), *Rankl* (**e**) and *Acp-5* (**f**) under low salt (LSD), normal salt (NSD) or high salt diet (HSD) in dental-periodontal tissue at the first upper molar, assessed with RT-qPCR (*n* = 8). Symbols represent single data points, horizontal lines the arithmetic mean and vertical lines the standard error of the mean. AU: arbitrary units, OTM: orthodontic tooth movement, LSD: low salt diet, NSD: normal salt diet, HSD: high salt diet. Statistics: ANOVA followed by Holm–Sidak multiple comparison tests. * *p* < 0.05; ** *p* < 0.01; *** *p* < 0.001.

To investigate the role of salt-containing diets on orthodontic tooth movement (OTM), we fed mice either a low salt diet (LSD), a normal salt diet (NSD) or a high salt diet and induced orthodontic tooth movement by insertion of an elastic band. First, we tested the impact of different salt contents and OTM on the expression of genes involved in bone remodeling processes. We detected no effects of OTM (LSD: *p* = 0.9999; NSD: *p* = 0.9612; HSD: *p* = 0.6282) on bone-mineralization-associated alkaline phosphatase (*Alpl*; Figure 2a). Different nutritional salt contents also had no effect on *Alpl* gene expression compared to a normal salt diet.

In line with this, we detected no changes in gene expression of *runt-related transcription factor 2* (*Runx-2*; LSD: *p* = 0.8280; NSD: *p* = 0.7515; HSD: *p* > 0.9999), which is a key transcription factor for osteoblast differentiation, induced by OTM. Salt treatment did not affect *Runx-2* gene expression in the periodontal ligament of mice (Figure 2b). *Prostaglandin endoperoxide synthase 2* (*Ptgs-2*) is involved in the synthesis of prostaglandin E2, which affects bone remodeling. We detected no effect of OTM on *Ptgs-2* gene expression upon LSD treatment (*p* = 0.9634), whereas orthodontic treatment significantly increased *Ptgs-2* gene expression under NSD (*p* = 0.0080) and HSD (*p* = 0.0019) conditions (Figure 2c). HSD elevated *Ptgs-2* gene expression with OTM compared to LSD (*p* = 0.0104) and NSD (*p* = 0.0143). Osteoprotegerin (*Opg*) acts as receptor activator of NF-kB ligand (*Rankl*) decoy receptor and is thereby critically involved in osteoclastogenesis [6,7]. We detected no effects of orthodontic treatment (LSD: *p* > 0.9999; NSD: *p* = 0.9983; HSD: *p* = 0.9626) or the different salt-containing diets on *Opg* gene expression (Figure 2e). In contrast, *Rankl* gene expression was elevated due to OTM (LSD: *p* = 0.0134; NSD: *p* = 0.0493; HSD: *p* < 0.0001) in all tested diets. HSD further increased *Rankl* gene expression compared to LSD (*p* = 0.0001) and NSD (*p* = 0.0015; Figure 2e), indicating increased osteoclastogenesis upon HSD. Accordingly, we detected enhanced *Acp-5* gene expression with NSD (*p* = 0.0041) and HSD (*p* < 0.0001) treatment, whereas this was not detectable with LSD (*p* = 0.7711; Figure 2f).

### 2.3. Effects of Different Salt Diets on Periodontal Bone Loss, Orthodontic Tooth Movement and Alveolar Bone Density

**Figure 3:**
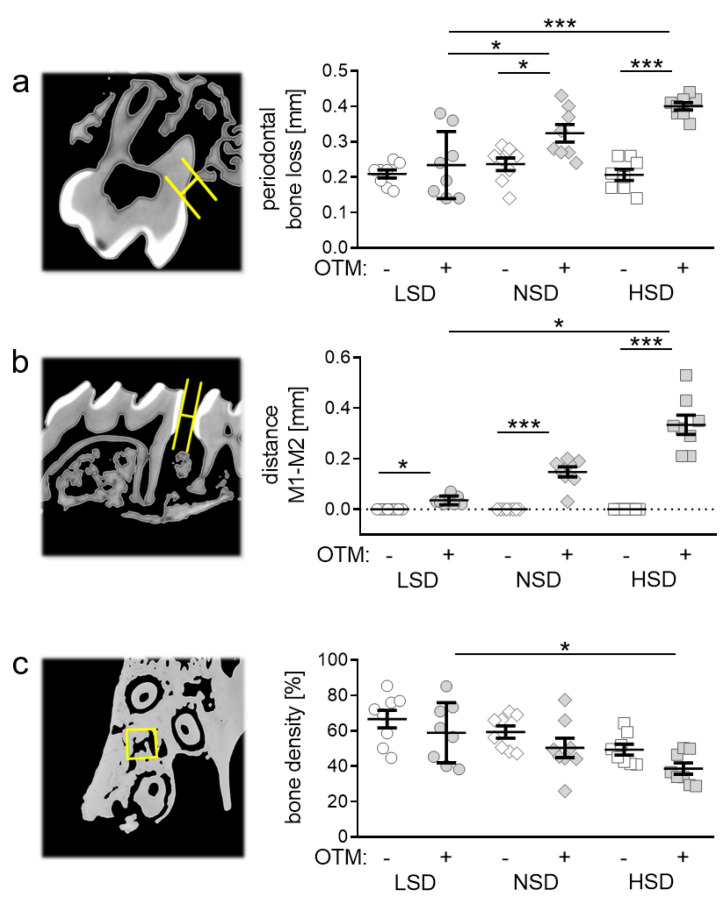
Analysis of periodontal bone loss (**a**), distance between the first (M1) and second (M2) upper molar (**b**) and bone density (**c**) after LSD, NSD or HSD, based on micro-computed tomography (µCT, *n* = 8). Symbols represent single data points, horizontal lines the arithmetic mean and vertical lines the standard error of the mean. AU: arbitrary units, OTM: orthodontic tooth movement, LSD: low salt diet, NSD: normal salt diet, HSD: high salt diet. Statistics: ANOVA followed by Holm–Sidak multiple comparison tests or Kruskal–Wallis test. * *p* < 0.05; *** *p* < 0.001.

Next, we investigated the impact of salt-containing diets on periodontal bone loss. We measured significantly enhanced periodontal bone loss due to OTM upon NSD (*p* = 0.0346) and HSD (*p* < 0.0001), but not with LSD (*p* = 0.8900; Figure 3a). NSD (*p* = 0.0308) and HSD (*p* < 0.0001) further elevated periodontal bone loss induced by orthodontic treatment compared to LSD (Figure 3a). OTM was determined by measuring the distance between the orthodontically moved first (M1) and the second (M2) upper left molar. With all diets we detected an increased distance between M1 and M2 after OTM (LSD: *p* = 0.0177; NSD: *p* = 0.0003; HSD: *p* < 0.0001; Figure 3b). HSD potentiated the extent of OTM compared to LSD significantly (*p* = 0.0179), leading to accelerated tooth movement (Figure 3b). To investigate the cause of this accelerated tooth movement, we investigated bone density between molars. We detected no significant effects of orthodontic treatment on bone density (LSD: *p* = 0.5196; NSD: *p* = 0.5196; HSD: *p* = 0.4724). HSD, however, reduced bone density compared to LSD with OTM treatment (*p* = 0.0251; Figure 3c), which could result in accelerated tooth movement.

### 2.4. Impact of the Osmoprotective Transcription Factor NFAT-5 on Osteoclast Activity

**Figure 4:**
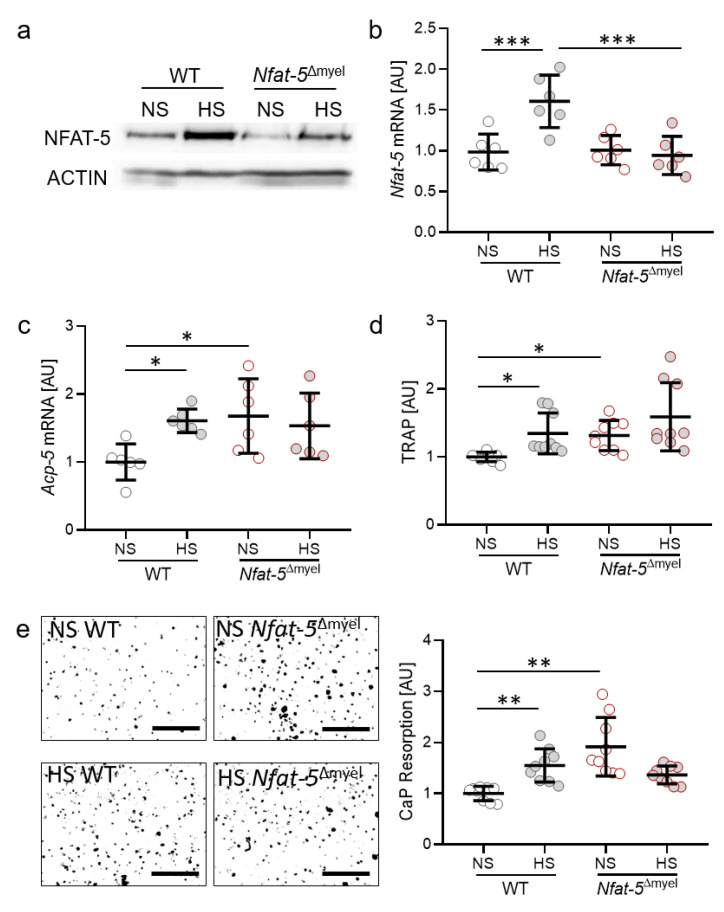
Protein (**a**) and gene expression (**b**) of NFAT-5. Gene expression of *Acp-5* (**c**), release of TRAP (**d**) and CaP resorption (**e**) with normal salt (NS) and high salt treatment (HS) in murine osteoclasts differentiated from bone-marrow-derived monocytes of wild-type or *Nfat-5*^Δmyel^ mice by treatment with M-CSF (30 ng/mL) and RANKL (50 ng/mL) for five days, followed by addition of 40 mM NaCl to the HS group (*n* ≥ 6). Symbols represent single data points, horizontal lines the arithmetic mean and vertical lines the standard error of the mean. AU: arbitrary units. Statistics: ANOVA followed by Holm–Sidak multiple comparison tests or Welch-corrected ANOVA with Games–Howell post-hoc tests. * *p* < 0.05; ** *p* < 0.01; *** *p* < 0.001.

The transcription factor NFAT-5 (nuclear factor of activated T cells 5) plays an important role in the osmoprotective adaption of cells and tissues to high salt conditions. Therefore, we investigated the role of NFAT-5 on osteoclast activity during salt treatment. Osteoclasts were differentiated from bone-marrow-derived monocytes (BMMs) derived from *Nfat-5*^Δmyel^ and control mice (WT). We determined increased NFAT-5 protein expression under HS conditions in osteoclasts from WT mice, whereas this effect was abolished in osteoclasts from *Nfat-5*^Δmyel^ mice (Figure 4a).

With reduced NFAT-5 expression in myeloid cells (Figure 4a), Osteoclasts differentiated from *Nfat-5*^Δmyel^-derived BMM failed to increase *Nfat-5* gene expression upon salt treatment (*p* = 0.0006; Figure 4b). We detected increased *Acp-5* gene expression with HS in WT mice (*p* = 0.0466; Figure 4c). Deletion of NFAT-5 in myeloid cells resulted in elevated *Acp-5* gene expression in NS conditions (*p* = 0.0317). Accordingly, we detected enhanced TRAP release in the cell culture supernatant (*p* = 0.0466) with HS in WT and with NS in *Nfat-5*^Δmyel^ (*p* = 0.0113; Figure 4d). In line with this, CaP resorption was elevated in *Nfat-5*^Δmyel^ osteoclasts (*p* = 0.0054; Figure 4e), indicating an inhibitory role of NFAT-5 on osteoclast activity.

### 2.5. Impact of NFAT-5 Deletion in Myeloid Cells on the Expression of Genes Involved in Bone Remodelling

**Figure 5:**
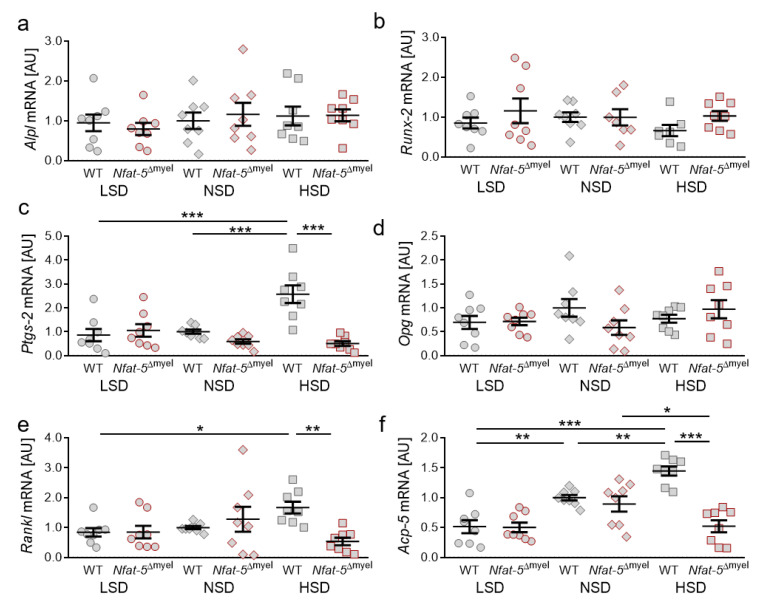
Gene expression of *Alpl* (**a**), *Runx-2* (**b**), *Ptgs-2* (**c**), *Opg* (**d**), *Rankl* (**e**) and *Acp-5* (**f**) with LSD, NSD or HSD in control or *Nfat-5*^Δmyel^ mice in dental-periodontal tissue at the first upper molar at the orthodontically treated side, assessed with RT-qPCR (*n* = 8). Symbols represent single data points, horizontal lines the arithmetic mean and vertical lines the standard error of the mean. AU: arbitrary units, OTM: orthodontic tooth movement, LSD: low salt diet, NSD: normal salt diet, HSD: high salt diet. Statistics: ANOVA followed by Holm–Sidak multiple comparison tests or Welch-corrected ANOVA with Games–Howell post-hoc tests. * *p* < 0.05; ** *p* < 0.01; *** *p* < 0.001.

Here, we analyzed the orthodontically treated sides of the jaw to evaluate the role of NFAT-5 on orthodontic tooth movement upon treatment with different salt-containing diets. We detected no effects of myeloid NFAT-5 deletion in *Alpl* (LSD: *p* = 0.9972; NSD: *p* = 0.9972; HSD: *p* = 0.9974; Figure 5a) or *Runx-2* gene expression (LSD: *p* = 0.8602; NSD: *p* = 0.9889; HSD: *p* = 0.8261; Figure 5b), indicating that myeloid-derived NFAT-5 plays no role in bone mineralization or osteoblast differentiation. *Ptgs-2* gene expression was elevated with HSD compared to LSD (*p* < 0.0001) and NSD (*p* ≤ 0.0001) in control mice (Figure 5c). This HSD-induced effect was reduced in *Nfat-5*^Δmyel^ mice (*p* < 0.0001; Figure 5c). We detected no effect of NFAT-5 deletion on *Opg* gene expression in the periodontal ligament (LSD: *p* = 0.9231; NSD: *p* = 0.3660; HSD: *p* = 0.8067; Figure 5d). In contrast, *Rankl* gene expression was upregulated upon HSD treatment in WT mice compared to LSD (*p* = 0.0429), whereas deletion of NFAT-5 in myeloid cells reduced this HSD-induced effect (*p* = 0.0042; Figure 5e). Last, we analyzed *Acp-5* gene expression and determined increased gene expression with HSD (*p* = 0.0095) compared to NSD, whereas LSD reduced *Acp-5* mRNA in WT mice (*p* = 0.0051; Figure 5f): Again, this HSD-induced effect was inhibited by NFAT-5 deletion in myeloid cells (*p* < 0.0001). Surprisingly, HSD reduced *Acp-5* gene expression in *Nfat-5*^Δmyel^ mice compared to NSD mice (*p* = 0.0289; Figure 5f).

### 2.6. Impact of NFAT-5 Deletion in Myeloid Cells on Periodontal Bone Loss, Orthodontic Tooth Movement and Alveolar Bone Density

**Figure 6:**
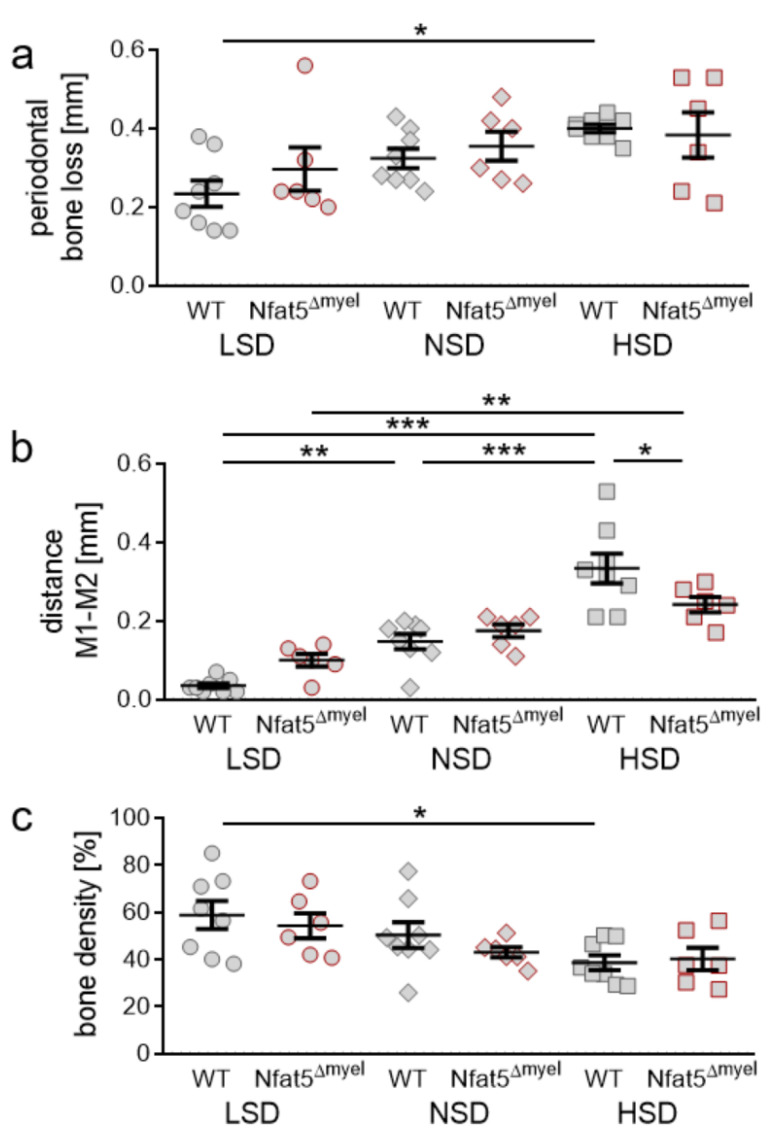
Analysis of periodontal bone loss (**a**), distance between the moved first (M1) and the second (M2) upper left molar (**b**) and bone density (**c**) after LSD, NSD or HSD in control or *Nfat-5*^Δmyel^ mice at the orthodontically treated side, based on µCT (*n* ≥ 6). Symbols represent single data points, horizontal lines the arithmetic mean and vertical lines the standard error of the mean. AU: arbitrary units, OTM: orthodontic tooth movement, LSD: low salt diet, NSD: normal salt diet, HSD: high salt diet. Statistics: ANOVA followed by Holm–Sidak multiple comparison tests or Welch-corrected ANOVA with Games–Howell post-hoc tests. * *p* < 0.05; ** *p* < 0.01; *** *p* < 0.001.

NFAT-5 deletion in myeloid cells had no impact on periodontal bone loss upon the investigated diets (LSD: *p* = 0.9149; NSD: *p* = 0.9770; HSD: *p* = 0.9996; Figure 6a). In *Nfat-5*^Δmyel^ we also detected increased OTM with HSD compared to LSD (*p* = 0.0018), but significantly less than in WT mice under HSD conditions (*p* = 0.0382; Figure 6b). NFAT-5 deletion did not affect alveolar bone density (LSD: *p* = 0.8843; NSD: *p* = 0.7600; HSD: *p* = 0.9114; Figure 6c). However, we detected no salt-induced reduction of bone density in *Nfat-5*^Δmyel^ mice compared to NSD (*p* = 0.9114) or LSD conditions (*p* = 0.5869; Figure 6c).

## 3. Discussion

In this study we observed increased osteoclast resorption activity upon salt treatment in vitro. In a murine model of tooth movement, increased *Rankl* and *Acp-5* gene expression were associated with reduced bone density, elevated periodontal bone loss and acceleration of orthodontically-induced tooth movement upon HSD. Furthermore, the underlying increased osteoclast activity was associated with osmoprotective transcription factor NFAT-5, as deletion in myeloid-derived cells resulted in enhanced osteoclast activity under NS conditions, whereas an induction in control mice failed under HS conditions. In vivo this impairment mainly manifested itself in the form of reduced *Rankl* and *Acp-5* gene expression and reduced orthodontic tooth movement (OTM) under HSD conditions in *Nfat-5*^Δmyel^ mice.

Salt-containing diets and other environmental challenges like inflammation have been associated with impaired sodium distributions in the body [16,20,26], promoting the reorganization of body metabolism [26,27]. Salt may affect osteoclast activity directly, as pumps acting as sodium exchangers, controlling the functionality of the hemi-vacuole involved in bone resorption [28,29,30]. In cell culture, however, the addition of sodium chloride is strictly associated with increased osmolality in the cell culture supernatant. Therefore, at this point of investigation, there is a possibility that the observed sodium-induced results could be due to changes in osmolality, which requires further research in this area.

One key regulator of OTM is cells of the periodontal ligament, such as fibroblasts or immune cells, which regulate the extent of bone remodeling by producing inflammatory cytokines and proteins affecting osteoclastogenesis, thereby modulating OTM [2,10]. It is known that periodontal ligament fibroblasts react to additional salt with increased prostaglandin E2 and RANKL expression [18,31], which could explain our results concerning *Rankl* gene expression at the orthodontically treated jaw side under HSD in the murine model. Furthermore, it has been reported that salt affects the expression of genes involved in extracellular matrix remodeling and inflammatory responses and thereby influences the reorganization of the periodontal ligament [18].

Previous studies investigating the role of salt on osteoclastogenesis of murine osteoclast progenitor cells revealed a tremendous impact, as the addition of 40 mM salt prevented the differentiation of osteoclast progenitor cells to osteoclasts, whereas lower concentrations were reported to promote osteoclastogenesis [19,32]. In line with our data concerning the alveolar bone, bone density in the tibia was reduced with HSD in control mice, whereas this effect was not observed in *Nfat-5*^Δmyel^ mice [19]. The reduced bone density was caused by an increased number of osteoclasts, whereas the number of osteoblasts did not change with the diet [19]. The osmoprotective transcription factor NFAT-5 was shown to control the expression of bone-protective OPG in myeloid cells and osteoblasts [19]. However, we detected no changes in *Opg* gene expression in the periodontal ligament of *Nfat-5*^Δmyel^ mice.

Our data strongly indicate that salt-containing diets accelerate orthodontic tooth movement (OTM) and promote periodontal bone loss due to reduced bone density, which may be attributed to enhanced osteoclast activity. NFAT-5 seems to influence this reaction to HS, as we detected impaired OTM and osteoclast activity upon deletion. Dietary salt intake may affect the velocity of orthodontic tooth movement in patients. As according to the WHO, salt intake in Western societies is higher than recommended, and patients with high salt intake are expected to show increased osteoclastogenesis and tooth movement velocity. As periodontal bone loss has been observed to be a side effect of high-salt-containing diets, a reduction of salt intake during orthodontic treatment is recommendable in clinical practice based on the available data.

## 4. Materials and Methods

### 4.1. Cell Culture Experiments

Experiment 1: We cultured 10,000 RAW264.7 macrophages (400319, Cell Lines Service, Eppelheim, Germany) in 1 mL α-MEM (F0925, Biochrom, Berlin, Germany) supplemented with 10% fetal bovine serum (P30-3306, PAN-Biotech, Aidenbach, Germany), 1% L-glutamine (SH30034.01, GE-Healthcare, Chigaco, IL, USA) and 1% antibiotics/antimycotics (A5955, Sigma Aldrich, St. Louis, MO, USA). Differentiation of osteoclasts was induced by addition of 30 ng/mL macrophage-colony-stimulating factor (M-CSF, 576404, Biolegend, San Diega, CA, USA) and 50 ng/mL receptor activator of NF-kB ligand (RANKL, 577102, Biolegend, San Diega, CA, USA). Macrophages differentiated five days to osteoclasts. After that time, we either incubated the differentiated osteoclasts without additional salt (normal salt, NS) or with additional 40 mM sodium chloride (NaCl, high salt, HS; 1162241000, Sigma Aldrich; St. Louis, MO, USA) for another 24 h.

Experiment 2: We isolated bone marrow from the tibia and femur of eight- to ten-week-old male LysM^WT^Nfat-5^fl/fl^ (control) and LysM^Cre^Nfat-5^fl/fl^ mice (*Nfat-5*^Δmyel^) with PBS (14190094, Thermo Fisher Scientific, Waltham, MA, USA). After centrifugation at 1400 rpm for 5 min, erythrocytes were removed with 5 mL haemolysis buffer (0.15 M NH_4_Cl (A9434, Sigma Aldrich, St. Louis, MO, USA), 0.01 M KHCO_3_ (237205, Sigma Aldrich, St. Louis, MO, USA) and 0.1 mM EDTA pH 8.0 (324504, Sigma Aldrich, St. Louis, MO, USA)) for 5 min. After the addition of 10 mL PBS, cells were centrifuged at 1400 rpm for 5 min and cultivated in 10 mL α-MEM, supplemented with 10% FBS (P30-3306, PAN-Biotech, Aidenbach, Germany), 1% L-glutamine (SH30034.01, GE-Healthcare, Chigaco, IL, USA), 1% antibiotics/antimycotics (A5955, Sigma Aldrich, St. Louis, MO, USA) and 30 ng/mL M-CSF (576404, Biolegend, San Diega, CA, USA) for 4 h. Non-adherent cells were centrifuged at 1400 rpm for 5 min, counted and 5 × 10^6^ cells were seeded on a cell culture plate and incubated for 4 days in α-MEM (supplemented as described above, but with additional RANKL (50 ng/mL, 577102, Biolegend, San Diega, CA, USA). Bone-marrow-derived osteoclasts were seeded out for experiments in α-MEM (including all supplements). After a preincubation time of 24 h, we added either no salt (NS) or 40 mM NaCl (HS; 1162241000, Sigma Aldrich; St. Louis, MO, USA) for another 24 h.

### 4.2. Animal Experiments

All animal experiments were performed according to German law (55.2.2-2532-2-567, 25 January 2018, Government of Lower Franconia, Germany) in compliance with the ARRIVE guidelines. We kept 48 LysM^WT^Nfat5^fl/fl^ (control) and 42 LysM^Cre^Nfat5^fl/fl^ mice (*Nfat-5*^Δmyel^) male mice aged 6 weeks either on low salt (LSD; ≤0.03% NaCl food pellets (ssniff-Spezialdiäten, Soest, Germany) and tap water), normal salt (NSD; 0.05% NaCl food pellets (ssniff-Spezialdiäten, Soest, Germany) and tap water) or high salt diet (HSD; 4% NaCl food pellets (ssniff-Spezialdiäten, Soest, Germany) and 0.9% saline) for two weeks overall. After one week on the specific diet, we inserted an elastic band (0.3 mm, Inwaria, Trier, Germany) between the first (M1) and second molar (M2) of the right side of the upper jaw (OTM-side), as previously described [33]. The left side remained untreated (control-side). After an additional week, we killed the mice and either put upper jaws in 5% formalin for µCT analysis (*n* = 8 control and *n* = 6 *Nfat-5*^Δmyel^) or froze them immediately in liquid nitrogen for RNA analysis (*n* = 8 control and *n* = 8 *Nfat-5*^Δmyel^).

### 4.3. RNA Isolation from Cell Culture

We used 500 μL TriFast (30-2010, VWR International, Radnor, PA, USA) per sample, added 100 μL chloroform (1.02445.1000, VWR International, Radnor, PA, USA) and vortexed for 30 s, followed by an incubation for 15 min on ice. Samples were centrifuged for 15 min at 4 °C and 13,000 rpm. The supernatant was mixed with 500 μL isopropanol (20842.330, VWR International, Radnor, PA, USA). After incubation overnight at −80 °C, we centrifuged the samples for 30 min at 4 °C and 13,000 rpm. The supernatant was removed and the pellet was washed twice with 750 μL 80% EtOH (32205, Sigma Aldrich, St. Louis, MO, USA). The pellet was dried for 30 min and resuspended in 20 µL RNAse-free water (T143, Carl Roth, Karlsruhe, Germany).

### 4.4. RNA Isolation from Mouse Tissue and cDNA Synthesis

The Invitrogen PureLink RNA Mini Kit (12183018A, Thermo Fisher Scientific, Waltham, MA, USA) was used to extract RNA from dental-periodontal tissue at the first upper molar, according to the manufacturer’s instructions. RNA amounts were measured with the NanoPhotometer (N60, Implen, Munich, Germany) to use equal concentrations for cDNA synthesis. Reverse transcription reagents were prepared as a master mix consisting of 2 µL MMLV buffer (M531A, Promega, Madison, WI, USA), 0.5 µL oligo_dt_ (SO132, Thermo Fisher Scientific, Waltham, MA, USA), 0.5 µL random hexamer (SO142, Thermo Fisher Scientific, Waltham, MA, USA), 0.5 µL 10 mM dNTPs (L785.1/2, Carl Roth, Karlsruhe, Germany), 0.5 µL RNAse Inhibitor (EO0381, Thermo Fisher Scientific, Waltham, MA, USA) and 0.5 µL reverse transcriptase (M170B, Promega, Madison, WI, USA) per sample. Reverse transcription was performed in 1 h at 37 °C, followed by heat inactivation of the transcriptase for 2 min at 90 °C.

### 4.5. Quantitative Real-Time Polymerase Chain Reaction (RT-qPCR)

RT-qPCR was carried out for further analysis of the cDNA in duplets. Subsequently, 1.5 μL cDNA sample and 13.5 μL of primer mix were combined in 96-well PCR plates (712282, Biozym Scientific, Hessisch Oldendorf, Germany). The primer mix consisted of 0.375 µL forward and 0.375 µL reverse primer (Table 1), 7.5 µL SYBR Green Jumpstart (S4438, Sigma Aldrich, St. Louis, MO, USA) and 5.25 µL RNAse-free water (T143, Carl Roth, Karlsruhe, Germany) per well. The quantification was carried out in the Mastercycler^®^ realplex (Eppendorf, Hamburg, Germany). All primers were designed in accordance with the MIQE quality guidelines [34]. For normalization of the target genes, we used a combination of two validated reference genes (Table 1). The relative gene expression was calculated with as 2^−ΔCq^ [35], with ΔCq = Cq (target gene)—Cq (mean reference genes).

### 4.6. Tratrate-Resistant Acid Phosphatase (TRAP) Assay

The TRAP assay was performed with cell culture supernatants using a TRAP staining kit (PMC-AK04F-COS, Cosmo Bio, Tokyo, Japan), following the manufacturer’s instructions, and staining was quantified at 540 nm with an ELISA reader after 3 h at 37 °C. (Multiscan GO, Thermo Fisher Scientific, Waltham, MA, USA).

### 4.7. Western Blot

Proteins were isolated using 8 M urea (U5378, Sigma Aldrich, St. Louis, MO, USA), supplemented with proteinase inhibitors (87786, Thermo Fisher Scientific, Waltham, MA, USA) and concentrations were determined with RotiQuant (K015.3, Carl Roth, Karlsruhe, Germany). Equal protein amounts were loaded on 8% polyacrylamide gels. We transferred proteins onto a PVDF membrane (T830.1, Carl Roth, Karlsruhe, Germany) within 90 min at 90 V. Membranes were blocked for 1 h using 5% milk (T145.3, Carl Roth, Karlsruhe, Germany) in TBS-T before incubation with diluted primary antibodies (NFAT5: 1:1000, PA1-023, Thermo Fisher Scientific, Waltham, MA, US; ACTIN: 1:3000, E1C602, EnoGene, New York, NY, USA) overnight. After washing, membranes were incubated with the secondary antibody (1:5000; 611–1302, Rockland Immunochemicals, Gilbertsville, PA, USA) for 1 h at room temperature. After washing, membranes were incubated with Forte Western HRP substrate (WBLUR0100, Sigma Aldrich, St. Louis, MO, USA) and signals were determined using VWR Genoplex (VWR International, Radnor, PA, USA).

### 4.8. Calcium Phosphate (CaP) Resorption Assay

For the coating of the calcium phosphate cell culture plates, a 2.5-fold simulated body fluid (SBF) solution (50% Tris buffer pH7.4 (T1503, Sigma Aldrich, St. Louis, MO, USA); 25% calcium stock solution (0.92 g CaCl_2_·2H_2_O (C5080, Sigma Aldrich, St. Louis, MO, USA); 20 g NaCl (3957.1, Carl Roth, Karlsruhe); 0.76 g MgCl_2_ (M2670, Sigma Aldrich, St. Louis, MO, USA); dissolved in Tris buffer pH 7.4) and a 25% phosphate-stock solution (0.49 g Na_2_HPO_4_ (P030.1, Carl Roth, Karlsruhe, Germany); as well as 0.88 g NaHCO_3_ (8551.1, Carl Roth, Karlsruhe, Germany); dissolved in Tris buffer pH 7.4) were prepared and sterilized. Approximately 1 mL was transferred to a conventional 12-well-plate and incubated at room temperature for three days [36]. The solution was changed to a freshly prepared CaP solution pH 7.4 (4.1 mL 1 M HCl (X942.1, Carl Roth, Karlsruhe, Germany); 80 mL H_2_O_dd_; 0.05 g Na_2_HPO_4_ (P030.1, Carl Roth, Karlsruhe, Germany); 0.06 g CaCl_2_·2H_2_O (C5080, Sigma Aldrich, St. Louis, MO, USA); 0.82 g NaCl (3957, Carl Roth, Karlsruhe, Germany); 0.61 g Tris-buffer (T1503, Sigma Aldrich, St. Louis, MO, USA)), incubated at room temperature for one additional day. Removal of the CaP solution was followed by addition of 800 µL 70% EtOH (32205, Sigma Aldrich, St. Louis, MO, USA) per well. The plates were washed twice with sterile water (L0015; Biochrom, Berlin, Germany) and dried. To determine the resorption activity of calcium phosphate, the supernatant was removed and cells were washed with 1 mL prewarmed PBS twice, following addition of 200 μL of a 1 M NaCl (3957.1, Carl Roth, Karlsruhe, Germany), mixed with 0.2% Triton (T9284, Sigma Aldrich, St. Louis, MO, USA) for 10 min. The coated wells were washed twice with water to remove cells from the surface. This was followed by the addition of 200 µL 5% AgNO_3_ (7908.1, Carl Roth, Karlsruhe, Germany) per well. The coated wells were illuminated with UV light at room temperature for 45 min and washed with water. They were photographed using a light microscope and resorption lacunae were evaluated using ImageJ software (ver. 1.47, Wayne Rasband, National Institutes of Health, Bethesda, MD, USA).

### 4.9. Micro-Computed Tomography (µCT)-Analysis

The upper jaw samples were kept in 5% formalin for 24 h, then formalin was diluted to 0.1% until µCT measurements. Measurements were performed with the Phoenix vltomelxs 240/180 device (GE Healthcare, Chicago, IL, USA), using the 180 kV NF tube under the following settings: voxel size: 10 µm, images: 1800, timing: 333 ms, voltage: 50 kV, current: 750 µA, Fastscan: Scan time 10 min. Image reconstruction and evaluation were performed using the software VG Studio Max (Volume Graphics, Heidelberg, Germany). A two-dimensional plane was selected for the various measurements running through the middle of the first molar at the level of the enamel-cementum boundary and aligned perpendicular to the palate and the chewing plane to enable reproducible measurements. Periodontal bone loss around the first molars was determined orally. For this purpose, the distance between the enamel-cementum boundary and the bony limbus alveolaris on the treated and the control side was measured. To determine orthodontic tooth movement (OTM), we measured the distance between the moved first (M1) and the second (M2) upper left molar. We determined the smallest distance between the crowns of M1 and M2 using the caliper function of the software. The measurement was carried out both on the orthodontically treated side and on the control side. To determine alveolar bone density, an interradicular, cube-shaped (edge length 0.35 mm) region of interest (ROI) was analyzed using the morphometric function of the software. Care was taken to ensure that this ROI did not reach into or overlap the periodontal ligament or the tooth roots.

### 4.10. Statistical Analysis

Normal distribution was tested with Shapiro–Wilk tests. Comparing two groups, Student’s *t*-tests were performed. Analyzing more than two groups, either ordinary ANOVAs followed by the Holm–Sidak multiple comparison tests or Welch-corrected ANOVAs followed by Games–Howell multiple comparison tests were performed. The distance between M1 and M2 was analyzed using the Kruskal–Wallis test. Differences were considered significant at *p* < 0.05. Statistical analysis was performed with GraphPad Prism version 8.0 (GraphPad Software, San Diego, CA, USA).

## Figures and Tables

**Table 1:** Sequences of used reference and target genes. The combination of *Eefa1a/Ywhaz* was used for Figure 2. *Sdha/Ywhaz* was used for osteoclast-derived samples.

Symbol	Gene Name	Number	5′-Forward Primer-3′	5′-Reverse Primer-3′
*Acp-5*	acid phosphatase-5, tartrate resistant	NM_007388.3	ATACGGGGTCACTGCCTACC	TCGTTGATGTCGCACAGAGG
*Alpl*	alkaline phosphatase, tartrate resistant	NM_007431.3	GGGGTACAAGGCTAGATGGC	AGTTCAGTGCGGTTCCAGAC
*Ptgs-2*	prostaglandin endoperoxide synthase-2	NM_011198.4	TCCCTGAAGCCGTACACATC	TCCCCAAAGATAGCATCTGGAC
*Eef1a1*	eukaryotic translation elongation factor 1 alpha 1	NM_010106.2	AAAACATGATTACAGGCACATCCC	GCCCGTTCTTGGAGATACCAG
*Nfat-5*	nuclear factor of activated T cells-5	NM_133957.3	AAATGACCTGTAGTTCTCTGCTTC	GCTGTCGGTGACTGAGGTAG
*Polr2a*	polymerase (RNA) II (DNA directed) polypeptide A	NM_001291068.1	CGGATGGTGTGAGCCTGATG	GTTCTCGCTCCAGAGCCTTC
*Opg*	osteoprotegerin	NM_008764.3	CCTTGCCCTGACCACTCTTAT	CACACACTCGGTTGTGGGT
*Rankl*	receptor activator of NF-kB ligand	NM_011613.3	AAACGCAGATTTGCAGGACTC	CCCCACAATGTGTTGCAGTTC
*Runx-2*	runt related transcription factor-2	NM_009820.5	GACGTGCCCAGGCGTATTTC	CACCTGCCTGGCTCTTCTTAC
*Sdha*	succinate dehydrogenase complex, subunit A	NM_023281.1	AACACTGGAGGAAGCACACC	AGTAGGAGCGGATAGCAGGAG
*Ywhaz*	tyrosine 3-monooxygenase/tryptophan 5-monooxygenase activation protein, zeta	NM_011740.3	AATGCTTCGCAACCAGAAAGC	TGGTATGCTTGCTGTGACTGG

## Data Availability

All datasets are publically available either as supplementary information to this article or upon request from the corresponding author.
